# The effect of distance nurse-led fatigue management on fatigue, sleep quality, and self-efficacy in patients with multiple sclerosis: a quasi-experimental study

**DOI:** 10.1186/s12883-023-03115-8

**Published:** 2023-02-14

**Authors:** Mahla Qomi, Mahnaz Rakhshan, Mohsen Ebrahimi Monfared, Zahra Khademian

**Affiliations:** 1 Student Research Committee, School of Nursing and Midwifery, Shiraz University of Medical Sciences, Shiraz, Iran; 2 Community Based Psychiatric Care Research Center, School of Nursing and Midwifery, Shiraz University of Medical Sciences, Shiraz, Iran; 3 Department of Neurology, School of Medicine, Arak University of Medical Sciences, Arak, Iran; 4 Department of Nursing, School of Nursing and Midwifery, Shiraz University of Medical Sciences, Shiraz, Iran

**Keywords:** Fatigue, Multiple sclerosis, Nursing care, Patient care management, Self efficacy, Sleep quality, Telenursing

## Abstract

**Background:**

Fatigue is one of the most common problems in patients with multiple sclerosis (MS) and has adverse effects on their sleep status and self-efficacy. This study aimed to determine the effect of distance nurse-led fatigue management on fatigue, sleep quality, and self-efficacy in patients with MS.

**Methods:**

This quasi-experimental study was performed on 60 patients with MS in Arak, Iran. Subjects were randomly assigned into intervention and control groups. The intervention group received eight sessions of nurse-led fatigue management training through the Skyroom platform. The control group received only the usual programs. Data were collected before and two months after the intervention using the Fatigue Severity Scale, the Pittsburgh Sleep Quality Index, and the Multiple Sclerosis Self-Efficacy Scale. The significance level in this study was determined 0.05.

**Results:**

After the intervention, the mean score of fatigue severity in the intervention group was significantly lower than the control group (2.52 ± 0.40 vs 5.65 ± 0.52) (*P* < 0.001). Also, after the intervention, the mean score of self-efficacy in the intervention group was significantly higher than the control group (49.37 ± 3.25 vs 24.43 ± 2.52) (*P* < 0.001). Furthermore, after the intervention the mean score of sleep quality was lower in intervention group (11.92 ± 2.01) than the control group (15.46 ± 1.40) (*P* < 0.001).

**Conclusion:**

Distance nurse-led fatigue management improved fatigue, sleep quality, and self-efficacy in patients with MS. We recommend the use of these courses as an important step toward improving fatigue, sleep quality, and self-efficacy among these patients.

## Background

Multiple sclerosis (MS) is the most common progressive neurologic disease in young adults worldwide [1]. According to a report in 2020, about 2.8 million people worldwide are living with this disease [2]. Iran is a country in the Middle East with moderate to high prevalence of MS, ranging from 5.30 to 74.28 per 100,000 individuals [3, 4]. Fatigue is one of the most common symptoms of MS, ranged from 36.5 to 78.0%. It can significantly affect the patients' quality of life and impose an economic burden on the patients and healthcare delivery system. In addition, patients who experience fatigue are more likely to be unemployed or work less than patients without fatigue [5]. Treatment of fatigue is one of the priorities of MS patients. Pharmacological therapy, non-pharmacological treatment such as physical rehabilitation, physical activity and exercise therapy, and non-invasive brain stimulation are the measures taken to treat MS related fatigue [6, 7]. Furthermore, Psychological and behavioral interventions are among the treatments for fatigue associated with MS [6, 8].

Another problem reported by some MS patients is sleep disturbance. Studies confirm the high prevalence of poor sleep quality in patients with MS [9, 10]. The prevalence of sleep disorders among MS patients is 25–54% [11]. Poor quality of sleep in MS is associated with negative consequences such as reduced quality of life and increased fatigue [9, 10, 12]. Eliminating sleep disorders is known to be an effective way to relieve fatigue in these patients [13]. In addition, fatigue management intervention has improved the fatigue and sleep quality of people [14].

Self-efficacy is one of the impressionable psychological variables of MS, which affects the physical performance of patients with MS [15]. Based on Bandura's theory, self-efficacy is a psychological concept that refers to a person's degree of confidence in his/her ability to perform tasks and achieve goals in certain situations [16]. Self-efficacy is a predictor of health behavior and can play a role in improving the experience of a chronically ill patient. In patients with MS, low self-efficacy is associated with low health-related quality of life, less control over mood, and less social and physical activity [17]. In these patients, fatigue can affect the patients' daily performance and may cause them to question their belief in their self-efficacy [18].

Nurses play an important role in empowering patients to manage chronic diseases [19, 20]. They facilitate the management of complex diseases and improve lifestyle-related behaviors through nurse-led management interventions. In this way they communicate with the patients and provide feedback to them in performing care, based on accepted guidelines [21, 22]. Evidence has shown the positive effects of nurse-led strategies on sleep quality, fatigue, depression symptoms, and constipation in patients with cancer, fatigue and quality of life in patients with rheumatoid arthritis, and self-efficacy in patients with type 2 diabetes [14, 23–25]. We did not find any study that investigated the effect of nurse-led fatigue management on outcomes such as fatigue, sleep quality, and self-efficacy of patients with MS. However, it is necessary to use effective nursing interventions to improve these outcomes in patients with MS. Nurse-led interventions have shown positive effects among other patient groups [14, 26, 27]. Therefore, this study was conducted with the aim of determining the effect of distance nurse-led fatigue management on fatigue, sleep quality, and self-efficacy of patients with MS.

## Methods

This quasi-experimental study was conducted from March to August 2020 in the MS Association of Arak city, in the center of Iran. This charity association provides services including free and semi-free educational, therapeutic, and medical services for all patients. The Ethics Committee of Shiraz University of Medical Sciences approved the study.

### Participants

The number of participants was determined using MedCalc software, with α = 0.05, β = 0.2, Mean_1_ = 1.01, Mean_2_ = 1.42, S_1_ = 0.59, and S_2_ = 0.39. These means and standard deviations were extracted from the study of Zhang et al., which investigated the effect of a nurse-led home-based exercise and cognitive behavioral intervention on cancer-related fatigue [14]. Considering 20% attrition, the sample size was calculated to be 30 people in each group. The inclusion criteria in the current study were the age of 18–60 years, literacy and mastery in Persian language, the ability and facilities of using the Skyroom platform by the patient himself/herself or a member of his/her family, willingness to participate in the study, definite diagnosis of the disease by a specialist, being in remission phase of relapse-remitting type of MS, and passage of at least six months since the diagnosis of the disease. In addition, another criterion for entering the study was obtaining a fatigue score higher than 4 based on the fatigue severity scale. The exclusion criteria included absence in more than one training session; the individual's unwillingness to continue participation; a known case of cancer, anemia, thyroid diseases, orthopedic, and neurological diseases such as epilepsy, in which fatigue is one of the symptoms; and emergence of clinical conditions in the patient that lead to deterioration or death. Compensatory sessions were held for the participants who were absent in only one training session.

**Fig. 1:**
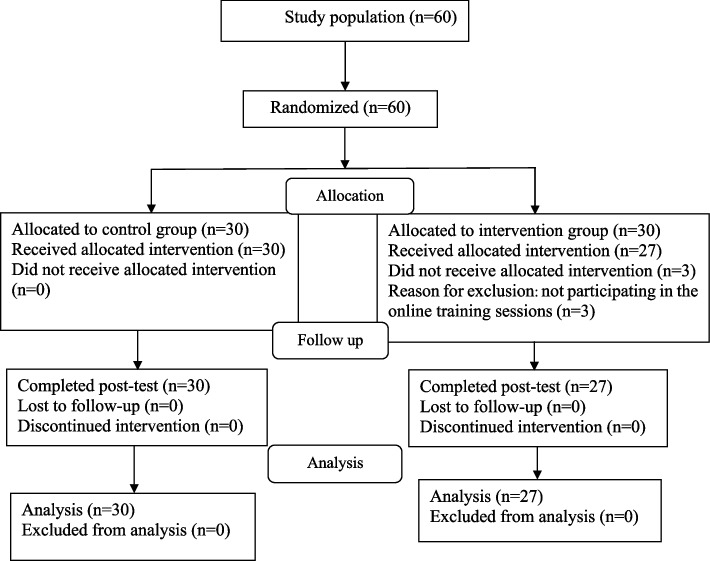
Study flow diagram. The diagram shows the progress of the participants throughout the study. After random allocation of patients to the intervention and control groups, three patients in the intervention group did not receive the allocated intervention. Finally, the data of 27 individuals in the intervention group and 30 individuals in the control group were analyzed

In the present study, for the purpose of sampling, the research assistant attended the MS association and provided the eligible patients who referred to the association with explanations about the research plan. The patients who agreed to participate in the study filled the informed consent form. Then, with block randomization (15 blocks of 4), they were placed in one of the two intervention and control groups. The selection order of the blocks was determined using the block randomization website. Finally, equal number of people were placed in each group. A total of 60 patients were included in the study. Three people from the intervention group were excluded from the study due to not participating in the online training sessions. Finally, the data of 57 patients (27 in the intervention group and 30 in the control group) were analyzed. The study flow diagram is presented in Fig. 1.

### Intervention

During the study, both the intervention and control groups received the routine care provided in the MS Association mentioned above. Moreover, the intervention group also received a nurse-led fatigue management program. This program consisted of eight training sessions of 60–90 min, which were provided through the native Skyroom platform. It is an Iranian platform that does not require the installation of any application, and users can enter the desired event with just a click. In this study, at first, the nurse-researcher sent a specific link to enter the class to each person in the intervention group, and they entered the class with their own username and password. One meeting was held every week, which included the topics of energy conservation and storage strategies, sleep hygiene, relaxing breathing exercises, and exercise at home (Table 1). In order to compile the content, we used similar studies and texts [14, 28, 29] and the opinion of experts (one neurologist, two nurses with PhD degrees, and one nurse with a bachelor's degree). The content included materials, photos and videos related to each topic, which were presented in the form of lectures and questions and answers. For the purpose of nurse-led management, the research nurse contacted the people of the intervention group a few days after each training session and evaluated the application of the materials taught to the patient. During the call, the problems that arose in the implementation of the educational content for the patient were raised, and with the cooperation of the patient, the available solutions were identified and implemented, so that the patient could make the most of the training according to his/her condition. In addition, in each phone call, the researcher encouraged the patient to adhere more to the provided training by providing appropriate feedback. The length of each call was approximately 10–15 min. In order to prevent the dissemination of educational materials to the control group, in addition to personalizing the link to enter the class through username and password for the people of the intervention group, they were also asked not to talk about the content with other patients. At the end of the study, the materials taught were given to both the intervention and control groups in the form of CDs and training booklets.

**Table 1:** Nurse-led fatigue management training program

1^st^ and 2^nd^ session	Energy maintenance and storage: prioritization and the correct way to perform activities, introduction of factors involved in fatigue and their correction, providing energy storage solutions
3^rd^ and 4^th^ session	Breathing exercises: teaching relaxing breathing and yoga breathing techniques
5^th^ and 6^th^ session	Sleep hygiene: training sleep mechanism and sleep–wake cycles, underlying and aggravating factors of insomnia, sleep hygiene protocol and proper sleeping methods
7^th^ and 8^th^ session	Exercise at home: teaching aerobic exercises, Pilates exercises, and stretching exercises at home for patients with MS

### Data collection

Patients completed self-report questionnaires online and individually before and eight weeks after the intervention. Data collection tools included general information questionnaire, and Persian versions of Fatigue Severity Scale (FSS), Pittsburgh Sleep Quality Index (PSQI), and Multiple Sclerosis Self-Efficacy Scale (MSSES).

#### General information questionnaire

General information questionnaire included demographic information and disease-related information. Demographic information included age, gender, marital status, education level, and employment status. The information related to the disease also included the duration of the disease, the number of hospitalizations, and comorbidities.

#### Fatigue severity scale

Krupp et al. designed the FSS in 1989 to measure fatigue in people with MS. The main fatigue intensity scale is a 9-item questionnaire, each part of which contains statements rated on a seven-point Likert scale from 1 "strongly disagree" to 7 "strongly agree". The total score of the scale is obtained from the average score of the statements and varies from 1 to 7. An average score of more than 4 is defined as fatigue. The validity of the tool showed that the FSS in patients with MS had a significant correlation with the visual pain scale (*P* < 0.01; r = 0.68). The reliability of the tool was obtained through Cronbach's alpha of 0.81. Moreover, the correlation coefficient of the score of each statement with the total score was reported 0.84 (*P* < 0.01) [30]. In the study of Shahvarughi-Farahani et al. (2013), the results showed that the Persian version of the FSS had a high correlation with the vitality subscale of SF-36 (*P* = 0.0001; r = -0.69) and the correlation coefficient of FSS with other subscales of SF-36 was between -0.65 and -0.43 (*P* = 0.0001). Its reliability was confirmed through Cronbach's alpha coefficient of 0.96 and ICC of 0.93 [31].

#### Pittsburgh sleep quality index

This tool was developed by Buysse et al. in 1998 to examine the sleep quality and help identify people with good or poor sleep quality in the general population. This questionnaire contains 18 questions in 7 components. The score of each component is a minimum of 0 and maximum of 3. The sum of the scores of these seven components constitutes the total score of the tool, which ranges from 0 to 21. A score higher than 5 indicates a severe problem in at least two fields or a moderate problem in at least three fields of the questionnaire items. In the study of Buysse et al., the instrument had a sensitivity of 89.6% and specificity of 86.5% in differentiating individuals who sleep well and individuals who sleep poorly. The reliability of PSQI was determined through the test–retest correlation coefficient of 0.85 (*P* < 0.001), and the internal consistency was confirmed through the Cronbach's alpha of 0.83 [32]. In the study of Farrahi Moghaddam et al. (2012), the Persian version of PSQI showed a significant correlation with the general health questionnaire (r = 0.54, *p* < 0.001), and its reliability was obtained through Cronbach's alpha of 0.77 [33].

#### MS patient self-efficacy scale

This scale was developed by Rigby et al. in 2003 in England to assess the self-efficacy of adult patients with MS. It is a multi-dimensional and self-report instrument developed with 14 items. The scoring of this scale is from completely disagree = 1 to completely agree = 6. The range of scores varies from 14 to 84, and higher scores mean higher self-efficacy. In the study of Rigby et al., the correlation between the score of the participants in this tool and the Schwartz general self-efficacy scale was reported 0.64, which indicates the validity of this tool. The reliability of this tool was confirmed through test–retest (r = 0.081, *P* < 0.001) and Cronbach's alpha coefficient of 0.81 [34]. In the study by Tanhaye Rashvanloo and Soleymanian [35] by removing three questions from the Persian version of the questionnaire, its reliability was calculated through Cronbach's alpha coefficient of 0.90 and Gottman's coefficient of 0.87. It indicates that the MS self-efficacy scale with 11 statements and a range of scores from 11 to 66 has suitable psychometric properties in the Iranian patient population [35]. In the current study, the instrument with 11 items was used.

### Data Analysis

Data analysis was done using SPSS software version 23. To evaluate the normality of the data distribution, we used the Kolmogorov–Smirnov test. Chi-square test was used to compare two groups based on qualitative variables. Also, paired t-test and Wilcoxon test were used for within-group comparisons, and Mann–Whitney U test was used to compare other quantitative variables between the groups. Moreover, analysis of covariance (ANCOVA) was used to eliminate the effect of age and education level on the findings. Cohen's d effect size was calculated for within-group and between-group changes. The effect sizes of 0.8, 0.5 and 0.2 were considered large, medium, and weak, respectively [36]. *P*-value less than 0.05 was considered statistically significant.

## Results

Most of the participants were in the age range of 31- 40 years (43.9%). Most of them were women (70.2%), married (66.7%), housewives (40.4%) and had diploma and post-diploma education (45.6%). Most of the participants had MS for 4 to 5 years (31.6%), had been hospitalized twice (31.6%), and had no comorbidities (70.2%) (Table 2).

**Table 2:** Comparison of general variables between the intervention and control groups

Variable	Total	Intervention (*n* = 27)	Control (*n* = 30)	*P* value*
	*n*(%)	*n*(%)	*n*(%)	
**Age range**				0.3
21–30	17 (29.8)	10 (37)	7 (23.3)	
31–40	25 (43.9)	9 (33.3)	16 (53.3)	
41–50	15 (26.3)	8 (29.6)	7 (23.3)	
**Sex**				0.259
Male	17 (29.8)	10 (37)	7 (23.3)	
Female	40 (70.3)	17 (63)	23 (76.7)	
**Marital status**				1
Single	19 (33.3)	9 (33.3)	10 (33.3)	
Married	38 (66.7)	18 (66.7)	20 (66.7)	
**Education**				0.067
High school	9 (15.8)	2 (7.4)	7 (23.3)	
Diploma and associate degree	26 (45.6)	12 (44.4)	14 (46.7)	
Bachelor	15 (26.3)	11 (40.7)	4 (13.3)	
Master's and higher	7 (12.3)	2 (7.4)	5 (16.7)	
**Employment status**				0.590
Employed	21 (36.8)	11 (40.7)	10 (33.3)	
Housewife	23 (40.4)	9 (33.3)	14 (46.7)	
Unemployed	13 (22.8)	7 (25.9)	6 (20)	
**MS duration**				0.666
6 months to 3 years	16 (28.1)	7 (25.9)	9 (30)	
4 to 5 years	18 (31.6)	10 (37)	8 (26.7)	
6 to 10 years	14 (24.6)	5 (18.5)	9 (30)	
11 to 15 years	9 (15.8)	5 (18.5)	4 (13.3)	
**Hospitalization frequency**				0.988
No hospitalized	9 (15.8)	4 (14.8)	5 (16.7)	
Once hospitalized	2 (3.5)	1 (3.7)	1 (3.3)	
Hospitalized twice	18 (31.6)	8 (28.6)	10 (33.3)	
Hospitalized three times	13 (22.8)	6 (22.2)	7 (23.3)	
Hospitalized more than three times	15 (26.3)	8 (29.6)	7 (23.3)	
**Comorbidity**				0.976
Yes	40 (70.2)	19 (70.4)	21 (70)	
No	17 (29.8)	8 (29.6)	9 (30)	

^*^Chi Square test

There was no significant difference between the intervention and control groups based on demographic and general variables (Table 2). However, due to the fact that practically, age and education level may have had an impact on the findings, ANCOVA was used to eliminate the effect of these demographic variables as covariates. The findings showed that by controlling the effect of these variables, the two groups had statistically significant differences in the mean post-test scores of fatigue severity, sleep quality and self-efficacy (*P* < 0.001). These findings show that education and age did not bias the findings.

The findings showed that after the intervention, the fatigue severity score of the intervention group was significantly lower than that of the control group (*P* < 0.001). Also, after the intervention, the fatigue severity score in the intervention group was significantly reduced compared to before the intervention (*P* = 0.002), while in the control group, the fatigue severity score increased after the intervention (*P* < 0.001). Also, after the intervention, a significant difference was observed between the self-efficacy score of the intervention group and the control group (*P* < 0.001). Moreover, the post-test self-efficacy score of the intervention group was significantly higher than the pre-test score (*P* < 0.001). In the control group, the patients’ self-efficacy decreased after the intervention (*P* = 0.001) (Table 3).

**Table 3:** Inter-group and within-group comparison of fatigue severity, sleep quality, and self-efficacy in the intervention and control groups before and after the intervention

**Time**		**Before intervention**		**After intervention**		***P***-value^*^	**Inter-group effect size**
**variable**	**group**	**Mean**	**SD**	**Mean**	**SD**		
Fatigue severity	**Intervention**	5.43	0.40	2.52	0.40	< 0.001	7.25
	**Control**	5.44	0.44	5.65	0.52	0.002	0.57
	*p*-value^**^	0.981		< 0.001			
	**Within-group effect size**	0.023		6.747			
Sleep quality	**Intervention**	15.42	1.62	11.92	2.01	< 0.001	2.16
	**Control**	15.45	1.41	15.46	1.40	0.955	0.07
	***p*-value**	0.942^**^		< 0.001^***^			
	**Within-group effect size**	0.019		2.043			
Self-efficacy	**Intervention**	25.59	3.36	49.37	3.25	< 0.001	7.19
	**Control**	25.50	3.10	24.43	2.52	0.001	0.47
	*p*-value^***^	0.872		< 0.001			
	**Within-group effect size**	0.026		8.576			

*Mann–Whitney U Test

**Paired t test

***Wilcoxon test

In addition, the post-test score of the sleep quality of the intervention group was significantly lower than the control group (*P* < 0.001). Also, after the intervention, the sleep quality score in the intervention group significantly reduced compared to before the intervention (*P* < 0.001), while the changes in the control group were not statistically significant (*P* = 0.955) (Table 3).

## Discussion

The findings of the study showed that the nurse-led fatigue management and the familiarity with fatigue reduction methods can improve fatigue, sleep quality, and self –efficacy of patients with MS. The high effect size values of the changes indicate that these improvements are clinically important. Evidence shows that few studies have examined the impact of the nurses-led fatigue management on sleep quality and patients’ self-efficacy. However, these interventions in other patient groups have shown positive effects [37, 38].

It is worth noting that patients who entered the current study had high fatigue scores at baseline. The findings showed that distance nurse-led fatigue management can reduce fatigue in patients with MS. Similarly, in a study of patients with ovarian cancer, nurse-led home based exercise, and cognitive behavioral therapy were effective in reducing the patients’ fatigue [14]. Of course, their study population and the number and gender of participants were different from our study. Moreover, in a study carried out in Switzerland, three weeks of energy management training in hospitalized patients suffering from fatigue caused by MS improved their self-efficacy in carrying out ergonomic behavioral changes and fatigue management strategies [39]. In their study compared to the present study, the duration of the disease was longer and the sample size was small. Contrary to our findings, in another study, energy storage management had no effect on the fatigue of patients with MS [40]. The reason for these differences can be the type of intervention. In our study, energy storage was part of the training provided, and other trainings to reduce fatigue and follow-up by phone could empower and motivate the patients to train and solve problems. In addition, the mean age of those involved in the previous study was 46—47 years which was older than those in our study. Furthermore, the duration of the disease in the previous study was 6.5- 7.5 that was less than that of our study. These also can be the reason for the differences. Moreover, the study of Paneroni et al. showed poor evidence of the effect of the exercises to reduce the fatigue of patients with chronic obstructive pulmonary disease [41]. Of course, the intervention used in the present study also addressed other aspects of fatigue management in addition to sports activities, which can justify the difference between these findings. It should be noted that, in this study we did not investigate the fatigue impact on patients' lives. Therefore, we recommend to address this issue in future studies.

Another finding of the study was that the distance nurse-led fatigue management could improve sleep quality of the patients. Similarly, in the study of Al-Sharman et al., aerobic exercises were effective on sleep quality and biological markers in patients with MS [42]. Similarly, another study showed an intervention that included sleep hygiene education, cognitive behavioral therapy, physical activity (e.g. 10–30 min of moderate aerobic exercises per week), and occupation therapy reduced fatigue and improved sleep quality of patients with MS [43]. Furthermore, a positive thinking program improved sleep quality of patients with thalassemia [44]. Based on the present findings, despite the improvement of sleep quality after the intervention, and considering the clinical significance of this finding, there was still a need to continue interventions to help patients achieve optimal sleep quality.

One of the other findings of the present study was the improvement of self-efficacy after participating in the distance nurse-led fatigue program. Similarly, in a study in Shanghai, China, a nurse-led phone follow-up education program based on self-efficacy was effective on the improvement of self -efficacy of patients with cardiovascular diseases [26]. Similarly, Shamsizadeh et al. showed that nurse-led telephone training and telephone follow-up had an impact on the self -efficacy of patients with type 2 diabetes [25]. However, a study in Toronto, Canada, showed that massage therapy did not affect the self-efficacy of patients with MS in the long run (eight weeks and more), and researchers suggested the use of continuous interventions [45]. Of course, the type of the disease was different in this study, and none of the patients had relapsing–remitting type of MS. Moreover, in the previous study massage therapy was performed by trained therapists, while in our study the massage was performed by the patient himself and, therefore, was applicable at any place and time.

Evidence shows that during COVID-19 pandemic, patients with MS experienced higher fatigue and weaker mental health indicators. In addition, they left their home less frequently and had less daily activities [46, 47]. Probably, for these reasons, the participants of this study used the services provided in the MS Association less than before. Perhaps because of these reasons, the fatigue and self-efficacy of control group who did not receive intervention worsened over time.

Based on our searches, the present study seems to be the first study on nurse-led fatigue management in patients with multiple sclerosis. Hence, the lessons learned from this research should be useful in designing and implementing future studies. One of the strengths of this study is the provision of non-attendance and distance training. This intervention provided the participants access to training they needed and there was no need to visit in person to receive training during COVID-19 pandemic. Hence, we recommend to emphasize the use of distance nursing for this group of patients. Another strength of this study is assigning the personal username and password for each participant to enter the Skyroom website, which reduced the possibility of sharing educational materials between the members of the intervention and control groups. In addition, patients in the intervention group were urged not to talk about the educational program with other patients. However, there is a possibility of information leakage between the two groups, which is one of the limitations of the study. In addition, due to the nature of our intervention, it was not possible to blind the participants, but in order to blind the researcher, pre- and post-intervention data were collected by a research assistant. Another limitation of this study was the short-term follow-up period. Therefore, the effectiveness of similar interventions should be examined for a longer period of time. One more limitation is that the attrition occurred only in the intervention group. Nevertheless, two groups were comparable based on general information and baseline main variables. Moreover, since quasi-experimental studies are subject to threats of internal validity, we recommend that the study design be improved in future research projects.

## Conclusion

The findings of this study showed that the distance nurse-led fatigue management can improve fatigue, sleep quality, and self -efficacy in patients with MS. These findings showed the important role of nurses in community-oriented care of patients with MS. Since nurses can play an effective role in the management of patients with MS, they should pay more attention to the patients’ fatigue. Moreover, it is necessary for them to learn fatigue reduction skills and include them in patient care protocols. We recommend teaching these skills to nursing students and including these skills in the nursing continuing education. In addition, we recommend the inclusion of nurse-led fatigue management as an inexpensive non-pharmacological intervention in the management of patients with MS. The distance nature of similar interventions provides the possibility of access to the people in remote areas, as well as providing services to patients in situations such as the COVID-19 pandemic. We recommend further research with a longer follow-up period on the effectiveness of similar interventions.

## Data Availability

Data resource and statistical analysis outputs can be provided by the corresponding author on reasonable request.

## Abbreviations

ANCOVA: Analysis of Covariance
COVID-19: Coronavirus Disease of 2019
MS: Multiple Sclerosis
MSSES: Multiple Sclerosis Self-Efficacy Scale
PSQI: Pittsburgh Sleep Quality Index
FSS: Fatigue Severity Scale
SPSS: Statistical Package for the Social Sciences
